# Artificial Intelligence in IPMN diagnosis: bridging promise and clinical reality

**DOI:** 10.1097/MS9.0000000000004170

**Published:** 2025-10-21

**Authors:** Sana Soomro, Eashaal Imtiaz, Maliha Khalid, Muhammad Talha, Aminath Waafira

**Affiliations:** a Department of Medicine, Jinnah Sindh Medical University, Karachi, Pakistan; b Department of Medicine, Ayub Medical College, Abbottabad, Pakistan; c King Edward Medical University, Lahore, Pakistan; d The Maldives National University, Malé, Maldives

**Keywords:** artificial intelligence, deep learning, intraductal papillary mucinous neoplasms, pancreatic cancer

## Abstract

Intraductal papillary mucinous neoplasms (IPMNs) are pancreatic cystic tumors with malignant potential, requiring accurate risk stratification for management. Current diagnostic reliance on imaging and clinical guidelines is limited by subjectivity and moderate sensitivity. Artificial intelligence (AI), particularly machine learning and deep learning models, has demonstrated significant promise in distinguishing benign from high-risk lesions using CT, MRI, and endoscopic ultrasound. Studies have reported diagnostic accuracies as high as 94–99.6%, outperforming traditional clinical assessments. Despite these advances, widespread implementation is hindered by lack of standardized imaging protocols, methodological transparency, and prospective multicenter validation. Integrating AI into clinical practice and international guidelines may enhance precision diagnostics and improve prognostic accuracy in IPMN management.

*To the Editor,*

Intraductal papillary mucinous neoplasms (IPMNs) are histologically defined as mucin-producing cystic tumors. They originate either within the main (MD-IPMN) or branch pancreatic ducts (BD-IPMN), inducing ductal dilation. IPMNs possess malignant potential and are capable of progressing into invasive carcinomas. IPMNs hold therapeutic importance; 50% of these pancreatic cystic lesions are discovered incidentally, and only 25% of the resected pancreatic tumors are actually IPMNs. To date, management decisions are made based on the Fakuoka guidelines, which suggest surgical resection for high-risk lesions and surveillance for low-risk cysts. A definitive diagnosis remains a limitation; however, Artificial Intelligence (AI) is being progressively employed by clinicians to differentiate benign cysts from high-risk lesions^[1]^.

AI, particularly through machine learning and deep learning models, has become a significant clinical adjunct in improving the diagnosis of IPMNs. Computed Tomography (CT) and Magnetic Resonance Imaging (MRI) scans are fed into these algorithms to extract distinct radiomic biomarkers that can predict malignant potential. A multicenter MRI-based study yielded a remarkable 81.9% diagnostic accuracy, surpassing the evaluation driven by conventional guidelines^[2]^. These models are calibrated using expansive multicenter data to detect image patterns that align with histopathological outcomes. Moreover, in retrospective endoscopic ultrasound (EUS)-based studies, these deep learning models demonstrated outstanding diagnostic efficacy(AUC 0.98; accuracy 94%), outperforming traditional clinician interpretation and standard EUS assessments^[3]^.

Modern research applying AI to IPMN imaging has revealed optimistic yet preliminary results. Quantitative imaging accurately distinguishes the results of two deep learning models, MRI and EUS imaging. These models demonstrated an AUC of 0.78 and 0.98, one showing that two features were independent variables for malignant IPMN with ORs ranging from 1.49 to 1.52 and 0.977 to 0.981^[4]^. Notably, the convolutional neural system built on EUS images provided exceptionally high accuracy (99.6%) in differentiating low-grade IPMNs from high-grade carcinoma/dysplasia, which exceeded the clinical guideline. The importance of further validating these findings by means of prospective trials, ensuring that the results are solid and generalizable across various patient populations, needs to be emphasized. The clinical incorporation of AI in the diagnosis of IPMNs is limited because of a lack of prospective validation and multi-site studies, so it is needed to confirm reliability before routine clinical use^[5]^.

Even with optimistic evidence, the use of AI in IPMN diagnosis still deals with major challenges. Currently, the main barrier to the diagnostic tools is the ability to identify the precursor of pancreatic cancer. Further barriers require better implementation of AI best practices and considerations, such as standardized workflows for radiomics, robust mechanisms for data balancing, and transparency in the reporting of methods used in studies, including image segmentation approaches. The major challenges include violations of AI best practices, radiomics workflow standardization, and clear reporting of study methodology, including segmentation and data balancing. Because of these limitations, AI tools are not introduced into international management guidelines^[6]^.

Recent progress in AI brings the promise of improvement of diagnostic accuracy concerning IPMNs; however, a persistent gap remains between experiment and clinical application. Much of the current evidence continues to be restrained by retrospective design, single-center methodology, and much variability in approach. Therefore, the end result leaves important questions unresolved about reliability and generalizability. Current ongoing discussion covers challenges such as limited prospective validation, the absence of standardized radiomics workflows, and inconsistent reporting practices. All these underscore that the incorporation of AI into internationally agreed management guidelines must be above just scores in accuracy metrics. Here, underlining the tension between innovation and clinical applicability puts a nuance to the literature, reframing AI not just as a breakthrough technology but as one that must present a good measure of validation and preparedness for transcendence to recognize its true worth^[7,8]^.

In conclusion, for AI to be adequately used in the diagnosis of IPMN, prospective investigations must highlight standardized imaging protocols and large multicenter clinical trials to establish applied significance. Additionally, using standardized reporting frameworks such as the Checklist for Artificial Intelligence in Medical Imaging is vital for achieving transparency and reproducibility. In order for AI tools to become part of international management guidelines, the advances must act on predicting the malignant transformation with greater accuracy and allow their routine application in clinical practice. This letter to the editor is in compliance with the TITAN guideline^[9]^.

## Data Availability

No data were generated for this manuscript.

## Research registration unique identifying number (UIN)

1. Name of the registry: NA. 2. Unique Identifying number or registration ID: NA. 3. Hyperlink to your specific registration (must be publicly accessible and will be checked): NA.

## Peer and provenance statement

This article was not commissioned and was externally peer-reviewed.
